# Mitchell's osteotomy in the management of hallux valgus: An Indian perspective

**DOI:** 10.4103/0019-5413.45327

**Published:** 2009

**Authors:** Asif Nazir Baba, Javid Ahmed Bhat, SD Paljor, Naseer Ahmed Mir, Suhail Majid

**Affiliations:** Department of Orthopaedics, SKIMS Medical College, Bemina, Srinagar, Jammu and Kashmir, India

**Keywords:** Bunion, hallux valgus, metatarsalgia, Mitchell's osteotomy

## Abstract

**Background:**

Hallux valgus is a common condition that affects the forefoot. A large number of procedures are described for managing this condition. Mitchell's osteotomy and its modifications are being widely used for treating hallux valgus. However, most of the studies describe the results from the developed world. We present results of the classic Mitchell's osteotomy in hallux valgus in Indian subcontinent.

**Materials and Methods:**

Forty eight adult patients (including 12 bilateral ones) in the age range of 18–60 years with hallux valgus were managed with the classic Mitchell's osteotomy. Pain over the bunion was the reason for surgery in 53 of 60 feet and cosmesis in the remaining 7 feet. Patients with hallux valgus angle more than 20° and not responding to a trial of conservative treatment were included. Patients having metatarsophalangeal (MTP) joint osteoarthritis (Grade II and higher), hallux rigidus, rheumatoid arthritis, and with subluxation of MTP joint were excluded from the study. Further, patients with first metatarsal more than 3 mm shorter than second metatarsal were also excluded.

**Results:**

The average follow-up period is 3 yrs (range 18months – 6yrs). About 55 feet (83%) were painless after surgery. Forty-two (70%) patients were happy with the cosmetic results of the surgery. Metatarsalgia was the reason for dissatisfaction with the procedure in five patients. The average correction of hallux valgus and the intermetatarsal angles achieved was 19.7° and 6.9°, respectively. Using the Broughton and Winson scoring system, 37 (61.7%) feet had excellent results, 18 (30%) had good, and five (8.3%) feet had a poor results.

**Conclusion:**

The classic Mitchell's procedure is a simple procedure and gives good cosmetic and radiological results.

## INTRODUCTION

Hallux valgus is characterized by medial displacement of the first metatarsophalangeal (MTP) joint with respect to great toe, with or without soft-tissue enlargement of the first MTP joint.^1^ It is predominantly seen in the shoe-wearing female population in their 4^th^, 5^th^, and 6^th^ decades.^2^ Sin-Fook and Hodgson^3^ found a 31% greater incidence in Chinese population who wore shoes versus those who did not. The condition is widely reported in the Western literature. The incidence of hallux valgus was as high as 50% in a study in South Africans^4^ and as low as 2% in a study on barefoot population.^5^ Surprisingly, there is a lack of literature on the incidence and the management of this condition in the Indian subcontinent.^6^

At present, more than 130 procedures are described for the correction of hallux valgus. These include soft-tissue procedures, distal metatarsal osteotomies, proximal metatarsal osteotomies, resection arthroplasty, and arthrodesis.

Mitchell's osteotomy, although first described by Hawkin,^7^ bears his name after he described his results on more than 400 such osteotomies.^8^ It is a double step-cut osteotomy through the neck of the first metatarsal, displacing the head fragment laterally and planterwards. Mitchell's osteotomy restores the load-bearing function of the feet to near normal.^9^ We describe our results of classic Mitchell osteotomy on 60 feet in 48 patients.

## MATERIALS AND METHODS

A hospital-based retrospective study of patients who underwent classic Mitchell's osteotomy for hallux valgus was conducted in the Kashmir region. The Kashmir region, having a population of more than 6 million, has severe winters and majority of the population wear shoes unlike other parts of India.

The patients who were operated between April 2001 and October 2005 and had completed a minimum follow-up of 18 months were included in study. The preoperative details were obtained from the medical records of the patients. The patients were followed up regularly in the out-patient department using a proforma developed for that purpose. All the patients included in the study had undergone surgery as a primary procedure. The inclusion criteria were patients with hallux valgus in the age of 18–60 years and with hallux valgus angle more than 20°. Patients presenting with pain as the primary complaint were given a trial of conservative treatment and those not responding underwent surgery. Patients having MTP joint osteoarthritis (Grade II and higher), hallux rigidus, rheumatoid arthritis, and with subluxation of MTP joint were excluded from the study. Furthermore, patients with first metatarsal more than 3 mm shorter than the second metatarsal were also excluded.

### Operative procedure

All the surgeries were done under tourniquet control. Dorsomedial incision was made over the bunion, avoiding the terminal branches of the medial division of superficial peroneal nerve. Y-shaped incision was made over the capsule and the capsule flap was raised from proximal to distal. Medial eminence was removed and two holes drilled in the metatarsal shaft, the distal hole being 1.5 cm proximal to the distal margin of the articular surface. Distal cut was made, leaving 3–6 mm of lateral shaft intact. The amount of intact shaft varied with the severity of the deformity. Proximally a complete osteotomy was done, 3–4 mm proximal to distal hole, and the intervening segment of bone removed. The distal fragment was shifted laterally and planterwards. This was the most significant step to prevent postoperative metatarsalgia. The osteotomy was then held by No. 1 absorbable sutures (Vicryl). Medial capsulorrhapy was done and the wound was closed.

**Figure 1:**
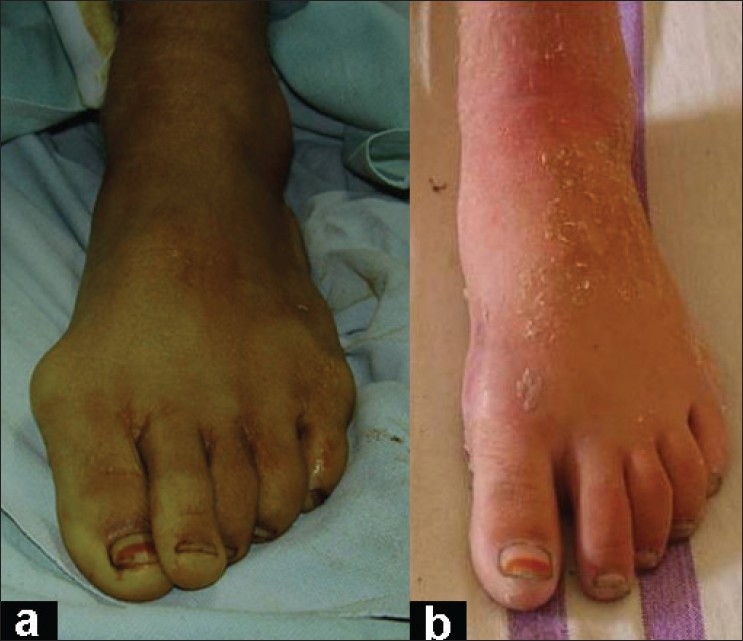
Clinical photograph (a) of left foot shows hallux valgus deformity, (b) Clinical photograph of the same patient shows correction achieved.

Following surgery, we gave a short plaster boot cast for two weeks. At two weeks, sutures were removed and a short leg cast was given with partial weight bearing allowed. Four weeks after surgery, full weight-bearing was started with a walking cast. The plaster cast was removed 8 weeks postoperatively, and full weight- bearing was encouraged immediately [Figure 1]. The patients were operated by different surgeons; however, the results were assessed by two of the authors (ANB, JAB).

All the patients underwent a detailed clinical examination with particular attention being paid to the presence of callosities, overlapping the second toe and other abnormalities in the forefoot. The patients were questioned about pain, cosmesis, and shoe-wear problems. Weight-bearing anteroposterior (AP) and lateral radiographs were recorded in all patients. Hallux valgus angle (angle subtended by lines bisecting the long axis of the first metatarsal and proximal phalanx), intermetatarsal angle (angle subtended by lines bisecting the longitudinal axis of the first and second metatarsals), first metatarsal length, and osteoarthritic changes were noted. After surgery, the patients were regularly followed up for a period ranging from 18 months to 6 years, the average follow-up being three years. The patients, on each follow-up, were questioned about the cosmetic appearance of foot, pain over the metatarsal head, shoe-wear problems, and shoe-wear modifications. Thorough clinical examination was done, looking for appearance, calluses under second and third metatarsal heads (transfer lesions), sensory abnormalities, and the range of motion of first MTP joint. Standing AP and lateral radiographs were studied for hallux valgus angle, intermetatarsal angle, osteoarthritic changes, and metatarsal shortening. Broughton and Winson scoring system^10^ as modified by Kartaglis^11^ was used to assess the final results [Table 1]. The result was regarded as excellent when the patient achieved Grade 1 in all categories, good when the patient had no more than two Grade 2s and no Grade 3s, and poor in any other case.

**Table 1:** Modified Broughton and Winson scoring system^11^

	Grade 1	Grade 2	Grade 3
Cosmetic appearance	Happy	Slight reservation	Unhappy
Pain in first MTP joint	None	Occasional	On normal activities
Metatarsalgia	None	After >3 h walking/standing	After < 3 h walking/standing
Function/activities	No restrictions	Slight restriction in daily activities	Severe restriction in daily activities
Shoe-wear	Any	Slight restriction	Difficulty in finding/only special shoes

## RESULTS

Fifty- seven patients with hallux valgus were operated using Mitchell's osteotomy. Nine patients were lost to follow-up and could not complete the minimum follow-up period of 18 months. The study describes the results of 60 feet in 48 patients comprising 44 female and four male patients. Twelve patients had a bilateral involvement. Thirty- seven patients belonged to rural area, and only 11 were from an urban background. The age of the patients ranged from 18 to 55 years, the average age being 35.7 years.

The most important indication for surgery was pain over the bunion. Forty- two patients (53 feet) reported pain over the first MTP joint as the primary reason for surgery. Six patients (7 feet) regarded cosmesis as the primary reason for surgery. Thirty- nine patients (47 feet) had some problem with shoe-wear, especially high-heeled shoes.

Fifty feet (83.3%) of 39 patients (81.25%) were painless after surgery. Seven feet (11.7%) of six patients (12.5%) had occasional pain and three feet (5%) continued to have pain even on normal activities. There was no case of worsening of pain and no patient who presented with cosmesis or shoe-wear restriction as primary symptom had pain after surgery. Forty- two patients (87.5%) (53 feet, 88.3%) were happy with the cosmetic appearance of the foot after surgery. Three patients (6.25%) (four feet, 6.7%) had some reservations regarding the cosmetic appearance of the feet, while three patients (6.25%) had recurrence of hallux valgus and hence were unhappy with the cosmetic results of the surgery.

Prior to surgery, 39 patients (47 feet) had some problem with footwear. After surgery, 36 patients (46 feet) could wear any type of shoe and the remaining 12 patients had problems with footwear. Of these, 10 patients (12 feet) still had some restriction in wearing all types of shoe-wear. This problem was more with wearing high-heeled shoes. Two patients had severe difficulty in shoes and required special shoes.

The average active dorsiflexion of the MTP joint after surgery as measured by goniometer was 41°, ranging from 15° to 65°. Ten feet had active dorsiflexion less than 30°, and all of them had some problem with wearing shoes, especially high-heeled ones. The average active postoperative planterflexion was 7° (range 0°–30°). However, no symptoms were attributed to this limitation in the planterflexion.

Postoperative metatarsalgia involving the second and occasionally the third or fourth metatarsal is an important reason for dissatisfaction with the procedure. Fifty- five feet (91.7%) of 43 patients had no complaint of metatarsalgia after surgery. Three feet (5%) had metatarsalgia only after walking or standing for more than 3 hours while two feet had metatarsalgia on standing or walking for less than 3 hours. Both the feet with severe metatarsalgia had metatarsal shortening of more than 10 mm of metatarsal shortening.

Thirty- five feet had callosity before surgery which improved in 23 feet. After surgery, no patient developed transfer lesion. Ten feet continued to have calluses but they were decreased in size and also had diminished pain. Two feet had no change in either size of calluses or decrease in pain over the callus.

**Figure 2:**
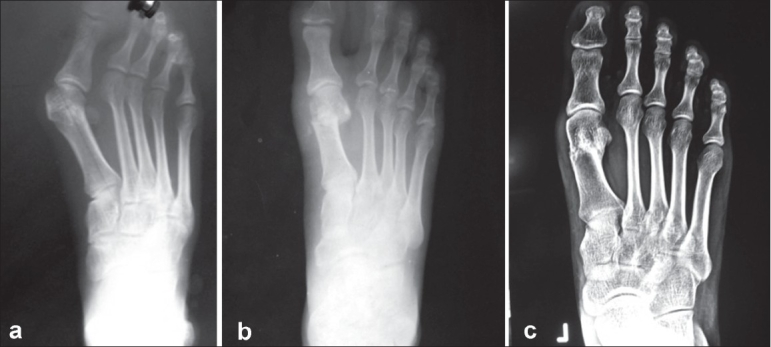
(a) Anteroposterior radiograph of left foot shows hallux valgus angle of 40°, (b) Radiograph at 4 months follow-up showing correction achieved, (c) Radiograph at 6 months showing the correction achieved and union at the osteotomy site.

Radiographs of the patients were examined closely. The average preoperative hallux valgus angle was 33.9° (range, 23°–55°). The average final hallux valgus angle was 14.2° (range, 3°–33°), with a correction of 19.7° (range, 3°–34°) being achieved [Figure 2]. The average intermetatarsal angle was 13.3° (range, 7°–22°) before surgery which got corrected to an average of 6.4° (range, 1°–13°). Thus, correction achieved in the intermetatarsal angle was 6.9° (range, 2°–16°). The surgery resulted in shortening of the first metatarsal and the average shortening of the first metatarsal produced following surgery was 5.4 mm (range, 0.2–10.4 mm).

At the end of the follow-up, which ranged from 18 months to six years, 56 feet had no restriction in function. Four feet had slight restriction in function and activities. No patient in our study had severe restriction in daily activities.

The most common complication of the surgery was decrease in the sensations over the medial aspect of the great toe, seen in seven feet (11.7%). This loss was transient in five feet, with two feet having persistent diminishment of sensations. This, however, did not have any influence on the patient's function or satisfaction with the procedure. Four feet (6.7%) had a recurrence of hallux valgus after achieving correction. All these patients had metatarsalgia and three of them were dissatisfied with the procedure. No patient had a hallux valgus after surgery. Four patients (four feet) had infection of the operative wound. The infection in all the cases was superficial and was managed with local dressings and antibiotics in all patients. Three feet developed swelling in the immediate postoperative period because of the tight cast. This was managed with release and repeat plaster after the subsidence of swelling. All these patients had good or excellent results. No patient in our series developed nonunion of the osteotomy site or avascular necrosis of the head of first metatarsal head.

Using the modified Broughton and Winson scoring system, 37 feet (61.7%) had excellent results, 18 feet (30%) had good results, and only five feet (8.3%) reported poor results. Thus, 91.7% feet had a satisfactory outcome after Mitchell's osteotomy for hallux valgus [Table 2].

**Table 2:** Final results using modified Broughton and Winson scoring system^11^

	Grade 1 feet	Grade 2 feet	Grade 3 feet
Cosmetic appearance	50	7	3
Pain in first MTP joint	53	4	3
Metatarsalgia	55	3	2
Function/activities	56	4	0
Shoe-wear	46	12	2

## DISCUSSION

Hallux valgus is the most common deformity affecting the forefoot. Hallux valgus angle greater than 15° and intermetatarsal angle more than 9° is regarded as hallux valgus.^14^ Forefoot being the most visible part of the foot, any deformity affecting the cosmesis of this part is bound to bring the patient to the surgeon. Pain over the medial eminence and problem in shoe wear (especially the high heel shoes) are the other major reasons for seeking treatment. The management of hallux valgus aims at a well-aligned and painless first MTP joint with the preservation of dorsiflexion, allowing normal progression in the gait cycle from foot-flat to toe-off stages.^13^ Till the late 19^th^ century, hallux valgus was managed conservatively. Reverdin^14^ is credited with designing the first procedure for the correction of this condition. Being so common, more than 130 procedures have been described for this condition.^15^ The goal of operative treatment is to offer relief of pain, correction of forefoot deformity and a bio-mechanically functional foot. Mitchell's osteotomy is widely used for the management of hallux valgus, especially in patients younger than 60 years with mild to moderate valgus deformity.

The major indication for Mitchell's osteotomy is painful bunion. Most (87%) of our patients presented with pain over the bunion as the primary complaint. In our study, 85% patients did not have any pain after surgery, and only 5% had pain even on normal activity. These results are comparable to other series that have reported satisfactory improvement in pain in 80–95% patients. In a study of 91 Mitchell osteotomies, in which painful bunion justified surgery in 92% of patients, Desjardins *et al.*^16^ achieved satisfactory improvement of pain in 92% of patients. Oye and Finsen^17^ had alleviation of pain in 35 patients of the total of 44 patients who underwent Mitchell's osteotomy. Dermon *et al.*,^18^ followed 51 feet with Mitchell's osteotomy for 10 years and had a satisfactory result in 90% patients. However, they noted a loss of 5° in the hallux valgus angle over the period.

Mitchell's osteotomy produces good cosmetic results. Most authors have reported satisfactory cosmetic outcome in more than 80% cases. Merkel^19^ followed 96 patients over a period of seven years and 86% of these patients were satisfied with the cosmetic outcome of the surgery. The patients were found equally in all age groups. Of the 60 feet undergoing Mitchell's osteotomy in our study followed for an average period of three years, 42 patients (87.5%) were happy with the cosmetic results. Only three patients were unhappy with the results of the surgery. All these patients had a recurrence of hallux valgus in the postoperative period. Glynn^20^ reported good or excellent results in 92% patients. Tan *et al.*^21^ followed 55 Mitchell's osteotomies and 92% of patients were satisfied with the results of the procedure. The patients stated that, given the identical situation, they would undergo the operation again.

We achieved an average correction of 19.7° in hallux valgus angle and 6.9° in the intermetatarsal angles with the average postoperative hallux valgus angle being 14.2° and intermetatarsal angle being 6.4°. Tan *et al.* achieved hallux valgus correction of 18.7° and intermetatarsal angle correction of 6.2°. Most of the authors have reported a correction of 10°–25°^20^,^22^–^24^ in hallux valgus and 5°–10°^9^,^22^–^24^ in intermetatarsal angles. Hallux valgus angle was more than 15° in 11 of our patients, including three feet with recurrence of the deformity. The rest of the patients were satisfied with the procedure as all of them had marked improvement in the hallux valgus angle. Establishing the normal anatomy of the foot is essential to prevent metatarsalgia.

In our study, we stabilised the osteotomy with heavy absorbable sutures. A number of modifications of the procedure have evolved.^25^ A number of studies have used metallic implants for holding the osteotomy. Modified Mitchell's osteotomy (Wu's bunienectomy)^26^ uses an oblique osteotomy with firm fixation with Herbert's screws. The reported advantages of using screws over suture are that it increases the strength and stability of the osteotomy site.^27^ Thus, it allows early weight-bearing and thereby decreases the period of immobilization and increases the range of motion (ROM) of the MTP joint.^28^ However, Calder^29^ in a study comparing the screw fixation with suture was not able to find any statistically significant difference between the two groups with respect to the pain score, patient assessment, or radiological measurement between the two. Further, there was no difference between the passive and active ROM at MTP joint at one year. The only advantage of using metallic implants is that it appears to encourage early return to work. Using suture for fixation of osteotomy site obviates the need for re-surgery for removing the implant. The use of sutures did not affect the stability of fixation as the recurrence rate of hallux valgus in our study is comparable to those that used metallic implants. Further, the use of metallic implants is associated with a high rate of complications related to the screw, with up to 15% patients needing screw removal by 6 months.

The most common reason for dissatisfaction with the procedure in most of the reported series, including ours, is the postoperative metatarsalgia involving the second metatarsal and sometimes the third and fourth metatarsals. Shortening of the first metacarpal is the main side effect of the procedure.^30^ Failure to adequately depress the metatarsal head and excess shortening of the first metatarsal is regarded as the major precursor to metatarsalgia. Merkel^19^ regards metatarsal shortening of more than 10 mm to be associated with metatarsalgia. However, a number of authors disagree with the concept of metatarsalgia being associated with first metatarsal shortening.^10^,^13^ We also found that metatarsal shortening correlates with metatarsalgia. All the patients in our study who reported postoperative metatarsalgia had shortening of 8 mm or more, and the two patients with severe metatarsalgia had shortening of more than 10 mm. To prevent the metatarsalgia Mitchell's osteotomy may be combined with oblique osteotomy of lesser metatarsals.^31^

We did not have any case of postoperative avascular necrosis of first metatarsal head. This is probably due to the preservation of soft tissues on the lateral side of the metatarsal. These structures on the lateral side are important for the blood supply of the distal fragment.^32^ Stiffness of first metatarsal was not a significant problem in our series. This is also attributed to less soft- tissue dissection on the lateral side of metatarsal.

Our overall results using the modified scoring system of Broughton and Winson^12^ showing a satisfactory outcome (excellent or good) in 92.3% feet correlates with the other reported results done in different parts of the world. Hart and Bentley^33^ compared five different procedures and found 78% satisfactory results with this technique. Mitchell (82%), Carr and Boyd^34^ (93%), Hammond^35^ (84%), Miller^36^ (90%), Glynn^20^ (92%), Blum^24^ (91%), and Tan^21^ (92%) all have reported good results.

## CONCLUSION

Mitchell's procedure is a technically simple procedure. The cosmetic and radiological results of the procedure are very satisfactory. The use of sutures to hold the osteotomy site is a time-tested and effective method and also prevents the need for a second surgery. Our series to best of our knowledge is the first reported series from Kashmir valley, and it is hoped that more such results are reported from the valley.
